# Vitamin D Knowledge, Attitudes and Practices of Polish Medical Doctors

**DOI:** 10.3390/nu13072443

**Published:** 2021-07-17

**Authors:** Wojciech Stefan Zgliczyński, Olga Maria Rostkowska, Beata Sarecka-Hujar

**Affiliations:** 1 Center of Postgraduate Medical Education, Department of Lifestyle Medicine, School of Public Health, 01-826 Warsaw, Poland; 2 Department of Transplantation Medicine, Nephrology and Internal Diseases, Medical University of Warsaw, 02-006 Warsaw, Poland; olga.rostkowska@wum.edu.pl; 3 Department of Basic Biomedical Science, Faculty of Pharmaceutical Sciences in Sosnowiec, Medical University of Silesia in Katowice, 41-200 Sosnowiec, Poland; bsarecka-hujar@sum.edu.pl

**Keywords:** vitamin D, supplementation, knowledge, attitudes

## Abstract

Background Vitamin D deficiency occurs in as much as 90–95% of the Polish population, although this condition is known to cause negative long-term health implications. The role of medical doctors in advising proper supplementation, monitoring and correcting the levels of 25-hydroxyvitamin D in individuals is of great importance and should be used to help mitigate its common deficits. The aim of this study was to evaluate knowledge, attitudes and practices of Polish physicians regarding vitamin D supplementation in order to identify areas for improvement and determinants for the knowledge gaps. Methods The study group comprised 701 medical doctors aged 32.1 ± 5.3 years on average, mostly women (71.61%). An original survey questionnaire was developed for the purpose of the study. Results The mean vitamin D knowledge score was 6.8 ± 2.3 (in a scale 0–13) and was related to gender (*p* < 0.001), type of specialization (*p* = 0.032), D3 supplements use (*p* < 0.001), recommending supplementation to patients (*p* = 0.005), to relatives and friends (*p* < 0.001) and to healthy adults (*p* < 0.001). In terms of self-administration, 14% of respondents take vitamin D all-year-round while 24% only in autumn and winter. 25% of respondents monitor their vitamin D (25-hydroxyvitamin D) serum concentration. Most participants (61%) did not recommend supplementing vitamin D to their patients on a regular basis. Conclusions The study indicates that medical doctors in Poland need to have more training and education on vitamin D supplementation in order to better address the problem of its deficits in the population.

## 1. Introduction

Vitamin D plays an essential role in a number of biological processes showing pleiotropic effect [1]. It reaches the body with food and is produced endogenously [2]. Vitamin D is synthesized in the human skin from 7-dehydrocholesterol in contact with ultraviolet light (UVB) but its production is limited by the availability of the substrate. Cutaneous synthesis of vitamin D can cover up to 80–100% of its daily requirement provided sufficient sun exposure [3]. To complement this process, dietary supplements of vitamin D are available in two forms: D3-cholecalciferol and D2–ergocalciferol [4]. In Poland, cholecalciferol is commonly used for prophylactic supplementation and treatment in case of hypovitaminosis [3]. The vitamin D status is found by measuring total plasma 25-hydroxyvitamin D [25(OH)D] concentration. The optimal level is over 30 ng/mL [5].

In Poland, almost 90% of the population was found to have deficits of vitamin D [3,6]. The study by Płudowski et al. [6], based on over 5700 Poles from 22 cities with mean age of 54.0 ± 15.9 years, demonstrated that in almost 66% of the study subjects 25(OH)D levels were lower than 20 ng/mL while suboptimal levels, from 20 to 30 ng/mL, were found in 24% of participants. Such deficits may be due to the geographic location and climate in Poland. The problem of vitamin D deficits in the general population occurs also in other Central European countries with insufficient sun exposure during the autumn and winter months [7,8].

Since vitamin D deficiency is common in each age group it became a global public health problem [8]. In the pediatric population, physicians are aware of rickets which is one consequence of the insufficient supply of vitamin D, while other symptoms such as movement disorder or nervous system abnormalities are less recognized [9]. A Chinese study on a group of 10,696 children and adolescents reported that 30% of them had vitamin D deficiency (<30 nmol/L), 80% demonstrated insufficient level of 25(OH)D (<50 nmol/L) while the measurements were lower for girls than boys [10]. In turn, in only 15.1% adults from northern Poland, 25(OH)D serum concentrations reached sufficient levels of ≥30 ng/mL [11]. Similarly, 75% of the elderly patients in Brazil had deficiency of vitamin D and low levels of 25(OH)D were found to strongly correlate with the risk of heart failure [12]. Other studies advocate for the role of vitamin D in developing metabolic and endocrine diseases, mental disorders or oncological conditions [13,14,15]. Therefore, maintaining optimal vitamin D supply plays a pivotal role in preventing the occurrence of many chronic health problems. 

The guidelines for vitamin D supplementation change every few years based on newest research. The latest version of the Polish recommendations for the prevention and treatment of vitamin D deficiency for the general population and for risk groups was released in 2018 [3]. Proper education of both patients and the medical community about the benefits of vitamin D and the importance of its proper provision according to the updated guidelines is of great importance.

The aim of the presented study was to evaluate knowledge, practices and attitudes of Polish medical doctors towards vitamin D supplementation. This study did not distinguish between types of vitamin D dietary supplements (D2 or D3).

## 2. Materials and Methods

### 2.1. Study Group

The study group comprised medical doctors and dentists participating in specialization courses at the Center of Postgraduate Medical Education in Warsaw, Poland from January to June 2019.

The presented study is not considered a medical experiment, so according to the Polish law the ethical approval is not required The survey used in this study was in line with the ethical standards of the institutional bioethical commission and the Declaration of Helsinki (1964). Although participation in the study was anonymous and voluntary, each surveyed participant expressed verbal consent.

### 2.2. Survey Questionnaire

The research tool was a self-administrated questionnaire developed for the purpose of this study in Polish. In preparation of the questionnaire previously published literature on this topic was analyzed.

The paper-based survey questionnaire contained 26 questions including: demographic characteristics of the respondent (gender, age, size of the place of residence), professional characteristics (main place of work, type and stage of specialization, advancement in the specialty training), health characteristics (BMI, consulting other doctors for one’s own health, number of hospitalizations in the past 3 years, medical treatments), self-supplementation of vitamin D (frequency, dosage, 25(OH)D serum concentration testing), counseling on the supplementation of vitamin D (to relatives and friends, patients, healthy adults and adults with confirmed deficiency), as well as questions that assess knowledge on vitamin D (sources of knowledge, causes of deficiency, groups that require supplementation, consequences of overdosing).

The questionnaire was piloted on a sample of 30 respondents working as medical doctors at the Department of Endocrinology, Centre of Postgraduate Medical Education in Warsaw, Poland. As a result of the pilot study, some questions have been modified.

A total of 701 correctly filled questionnaires were returned. The response rate was 82%. All questionnaires were completed correctly and included in the study.

### 2.3. Calculation of the Vitamin D Knowledge Score

Respondents’ knowledge of vitamin D was assessed in 4 questions concerning: causes of vitamin D deficiency (6 items), groups that particularly require vitamin D supplementation (5 items), and aspects related to the effects of vitamin D overdose (2 items).

Value 1 was assigned to each correct answer, and value 0 to each incorrect answer. Vitamin D knowledge score was constructed as a sum of scores for all answers. In total, it was possible to obtain a minimum of 0 points and a maximum of 13 points. The scale midpoint was 6.5. 

The following intervals of vitamin D knowledge score were analyzed: low score (0–4), moderate score (5–8), high score (9–13).

### 2.4. Statistical Methods

The statistical analyses were performed using STATISTICA 13.1 software (STATSOFT, Tulsa, OK, USA). The mean (M) and standard deviation (SD) were estimated for numerical variables, as well as absolute numbers (n) and percentage (%) of the occurrence of items for categorical variables.

Pearson’s chi-square test was used to correlate self-administration of vitamin D, self-testing of 25(OH)D concentration, recommending supplementation to relatives, patients, healthy adults and adults with diagnosed deficiency with such covariates as gender, 3 age groups, stage and type of specialization, having a Ph.D. and undergoing any treatment. All above-mentioned variables were categorical. If a significant difference was found, odds ratio (OR) was estimated.

Binary logistic regression analysis was used to profile the physician who does not prescribe vitamin D to his patients versus gender, age, type of specialization, whether a physician takes vitamin D personally, whether he advises it to his relatives and friends, to healthy adults, to adults with diagnosed deficiency.

Multivariate analysis of variance was used to correlate vitamin D knowledge score (a numerical variable) with categorical covariates such as: gender, 3 age groups, stage and type of specialization, having a Ph.D. and undergoing any treatment, as well as self-administration of vitamin D, self-testing of 25(OH)D serum concentration, recommending supplementation to relatives, patients, healthy adults and adults with diagnosed deficiency (categorical variables).

The significance level was assumed to be *p* < 0.05.

## 3. Results

### 3.1. Characteristics of the Study Group

Data was obtained from 701 medical doctors, aged 26–61, 32.1 ± 5.3 years on average, mostly women (71.6%). Analyzing 10-years age groups, 30–39-year-old respondents predominated in the study group (48.1%), followed by 20–29-year-olds (41.7%). The majority of respondents lived in cities over 500 thousand residents (58.4%). The demographic characteristics of the study group were presented in Table 1.

Professional characteristics of the study group were presented in Table 2. Respondents’ length of work as medical doctors ranged from 1 to 32 years, with average 5.7 ± 5.0 years. The main place of work for most respondents was a clinical hospital (49.9%) followed by a public hospital (36.5%). A total of 91.3% of respondents were during specialization, while 8.7% of respondents already completed at least one specialization. About 2/3 of respondents have already had or were during training in non-surgical specialization, 29%-surgical, 1%-both surgical and non-surgical. Approximately 7% of respondents had Ph.D.

Health characteristic of the study group were presented in Table 3. Most respondents had normal body mass (79.2%), used the advice of other doctors less frequently than once a year (49.1%), were not hospitalized in the past 3 years (74.0%), were not under any medical treatment (80.6%). One respondent had myocardial infarction or ischemic stroke in the past.

### 3.2. Attitudes towards Vitamin D Supplementation in the Study Group

About 19% of respondents have never taken any vitamin D supplements, while 44% took it occasionally, 24%-regularly in autumn and winter and 14%-regularly all-year-round (Table 4). Half of respondents took 2000 units of vitamin D per day, followed by 16% who took 1000 units.

About 25% of respondents tested their 25(OH)D serum concentration and 62% of them had deficiency.

Percentage of female doctors supplementing vitamin D (85.3%) was significantly higher than male doctors (70.9%), (*p* < 0.001). Moreover, percentage of women testing 25(OH)D serum concentration was two times higher than in case of men (28.1% vs. 14.7%, OR = 1.912, *p* < 0.001). 

Respondents with completed or ongoing non-surgical specialization declared vitamin D supplementation more often than those in the surgical path (82.8% vs. 75.7%, *p* = 0.035). Vitamin D was taken by 33.3% of respondents under medical treatment vs. 22.2% not undergoing any medical therapy (*p* = 0.007).

The prevalence of vitamin D supplementation did not differ significantly between 3 age groups (*p* = 0.210), between respondents with and without Ph.D. (*p* = 0.770), between respondents with completed specialization and those without it (*p* = 0.394), between respondents under medical treatment and not being treated (*p* = 0.694). Similarly, prevalence of 25(OH)D serum concentration testing did not differ significantly between 3 age groups (*p* = 0.149), between respondents with and without Ph.D. (*p* = 0.585), between respondents with completed specialization and those in training (*p* = 0.452), between respondents with surgical and non-surgical specializations (*p* = 0.109).

### 3.3. Practice in Vitamin D Supplementation in the Study Group

The majority of respondents recommended vitamin D supplementation to their relatives and friends (82.3%), their patients (81.4%), healthy adults (84.5%) and to adults with diagnosed vitamin D deficiency (89.2%) (Table 5).

Most doctors recommended vitamin D supplementation to the majority of their patients (39.1%). The most frequently chosen dose was 2000 units per day to healthy adults (it was recommended by 53.5% of respondents) and 4000 units per day to adults with diagnosed deficiency (recommended by 50.2%).

Female doctors recommended vitamin D supplementation statistically more often to relatives and friends (86.0% vs. 75.1%, *p* = 0.001), to patients (84.3% vs. 76.9%, *p* = 0.021) and to healthy adults (86.9% vs. 78.4%, *p* = 0.005) than male doctors. However, in case of recommending vitamin D to adults with known deficiency, there was no difference in terms of gender (96.2% vs. 93.2%, *p* = 0.214). 

Doctors with non-surgical specializations recommended vitamin D supplementation more often to patients (85.2% vs. 74.6%, *p* = 0.001) and to healthy adults (86.9% vs. 77.7%, *p* = 0.003) than doctors with surgical specialization. 

Prevalence of recommending vitamin D supplementation to relatives and friends, patients, healthy adults and adults with diagnosed deficiency did not differ significantly between 3 age groups (*p* = 0.235, *p* = 0.819, *p* = 0.309 and *p* = 0.838, respectively), between respondents with and without Ph.D. (*p* = 0.431, *p* = 0.865, *p* = 0.167 and *p* = 0.476, respectively), between respondents with specialization and those without it (*p* = 0.791, *p* = 0.548, *p* = 0.849 and *p* = 0.336, respectively), between respondents under medical treatment and not (*p* = 0.318, *p* = 0.983, *p* = 0.310 and *p* = 0.731, respectively).

Also, the prevalence of recommending vitamin D supplementation to relatives and friends as well as to adults with diagnosed deficiency did not differ significantly considering the type of specialization (*p* = 0.556 and *p* = 0.07, respectively).

A physician who does not prescribe vitamin D supplementation to his patients is significantly more commonly a man, with surgical specialization, not supplementing vitamin D personally, not recommending vitamin D supplements to relatives and friends, to healthy adults and to adults with diagnosed deficiency (Table 6).

### 3.4. Vitamin D Knowledge in the Study Group

Respondents gained knowledge about vitamin D from various sources. The most common sources were lectures or conferences (declared by 59.2% of respondents), followed by scientific articles (48.5%), medical handbooks (47.1%) and internet (33.7%). The least mentioned were pharmaceutical sales representatives (6.1%) and other medical doctors (1.1%).

Table 7 informs about the level of knowledge regarding vitamin D among study participants. The vast majority of respondents (97.2%) considered lifestyle, including spending time indoors, to be responsible for aggravating the vitamin D deficiency, 43.8% mentioned obesity, 33.5% common use of sunscreen with UV filter, 29.0% reduced intake of fats from diet, 26.3% air pollution, including smog, and 6.7% common use of statins. According to the respondents, groups that require vitamin D supplementation in particular are pregnant and lactating women (83.2%), people over 75 years of age (79.3%), children and adolescents up to 18 years of age (65.2%), overweight or obese individuals (54.9%) and young adults (26.5%). The majority of respondents (60.8%) agreed with a statement that vitamin D poisoning is a very rare complication of supplementation or treatment with cholecalciferol pharmaceutical preparations and in fact it only affects people with genetically determined hypersensitivity to vitamin D. Over 70% of the study participants knew that vitamin D poisoning is not a common complication of treatment with active vitamin D metabolites, e.g., alfacalcidol.

**Figure 1:**
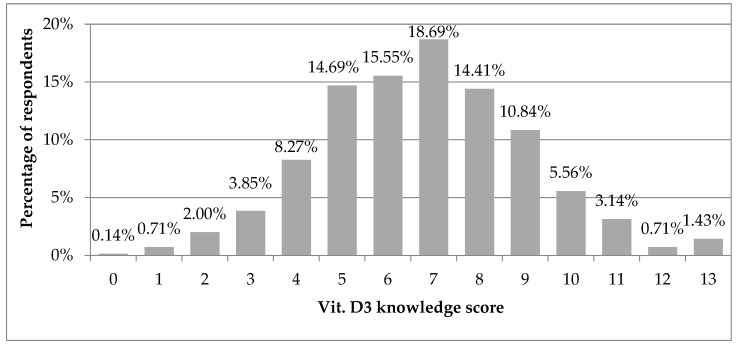
Vitamin D knowledge score in the study group (*N* = 701). Results are presented as %.

Vitamin D knowledge score was normally distributed with mean value of 6.8 ± 2.3, on a scale of 0–13. Analyzing each vitamin D knowledge score, most respondents (18.7%) obtained 7 points, which was the middle value (Figure 1). When analyzing the intervals of vitamin D knowledge score, 63.3% of respondents obtained moderate (5–8), 21.7%-high (9–13) and 15.0%-low values (0–4). Only 1.4% of respondents obtained 13 points, i.e., responded correctly to all questions. 

**Figure 2:**
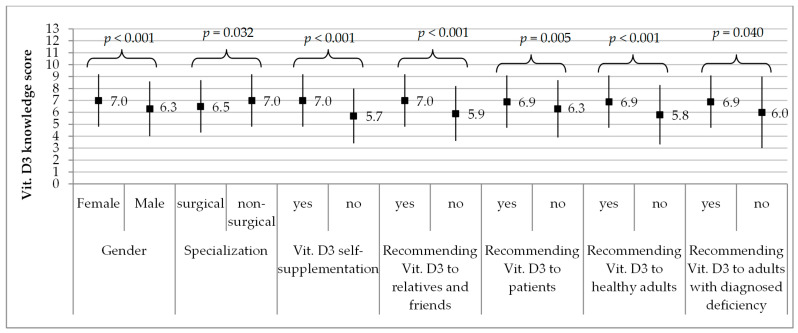
Vitamin D knowledge score against gender, type of specialization, vitamin D supplementation and recommending it to relatives and friends, patients, healthy adults, adults with diagnosed deficiency. Results are presented as M ± SD, M—mean, SD—standard deviation. Vit. D—vitamin D.

Significantly higher scores in vitamin D knowledge were obtained by females (7.0 ± 2.2 on average) vs. males (6.3 ± 2.3, *p* <0.001), respondents with completed or ongoing non-surgical specialization (7.0 ± 2.2) vs. those with surgical one (6.5 ± 2.2, *p* = 0.032), respondents supplementing vitamin D (7.0 ± 2.2) vs. those who do not (5.7 ± 2.3, *p* <0.001), and doctors who recommended supplementation of vitamin D to relatives and friends, patients, healthy adults and adults with confirmed deficiency vs. those who do not (Figure 2).

Vitamin D knowledge score did not differ significantly between 3 age groups (*p* = 0.661), between respondents with and without Ph.D. (*p* = 0.209), between respondents with completed specialization and without it (*p* = 0.814), between respondents undergoing medical treatment and those who do not (*p* = 0.637).

## 4. Discussion

We believe this is the first study evaluating the knowledge, attitudes, and practices concerning vitamin D supplementation among medical doctors in Poland. The study identifies knowledge gaps in this area, and indicates those groups of doctors to whom training should be addressed and tailored. 

Based on the obtained results, almost one fifth of medical doctors have never supplemented vitamin D while one seventh took it regularly throughout the whole year. Half of respondents took 2000 IU of vitamin D per day, while over one sixth took 1000 IU/day. According to the latest guidelines for the Polish population, vitamin D supplementation is not necessary, although recommended and safe, when healthy adults (19–65 years old) have enough daily sun exposition, i.e., bear uncovered forearms and legs without using sunscreen for at least 15 min a day between 10.00 am and 3.00 pm from May to September [3]. Otherwise, vitamin D should be supplemented in a dose from 800 to 2000 IU/day, based on body weight and dietary intake throughout the whole year.

Almost 50% of respondents in our study were 30 to 39 years old. Vitamin D deficiency was observed in 62% of medical doctors who had ever tested 25(OH)D serum concentration levels. The systematic review by Sowah et al. [16] demonstrated that the highest percentage of cases having vitamin D deficiency among healthcare professionals concerned healthcare students, then medical residents, followed by practicing physicians (72%, 65% and 46%, respectively). The authors observed that the mean level of 25(OH)D serum concentration of practicing physicians was 55.0 ± 5.8 nmol/L and was significantly lower in medical residents as well as healthcare students [16]. In a research paper by Haney et al., 74% of internal medicine residents had lower levels of 25(OH)D in the spring compared to autumn [17]. Interestingly, Munter et al. [18] who analyzed serum concentrations of 25(OH)D in a sample of medical doctors in Jerusalem observed that hospital physicians had significantly lower mean levels compared to community-based practitioners (15 ± 6 vs. 19.7 ± 6 ng/mL, respectively). The authors considered the level of 20 ng/mL to be the cutoff and noticed that vitamin D deficiency was found in 77% of hospital doctors vs. 68% of community doctors [19]. As much as 97% of physicians from a tertiary center in the north of India had vitamin D deficiency, which is likely due to the fact that only 10 out of 200 study subjects had proper sun exposure for more than 30 min/day [16]. In case of individuals with darker skin pigmentation, it is necessary to provide longer exposition to UVB than recommended for people with lighter skin types [20].

Almost 82% of the analyzed doctors supplemented vitamin D, more women than men (85.3% vs. 70.9%). It resonates with studies which confirm that female doctors are more likely to adhere to medical guidelines and recommended prevention strategies compared to male doctors [21,22]. On the other hand, some researchers suggest that female doctors are likely to prescribe more medicinal products than male doctors [23]. Studies dedicated to differences in self-supplementation of vitamins among doctors are scarce and should be further explored.

In our study, females tested 25(OH)D serum concentration significantly more often than males (28.1% vs. 14.7%) which is consistent with the findings by Rodd et al. [24] who observed that in the local population of Manitoba (Canada) women monitored their 25(OH)D level twice more frequently. At the same time, the authors observed a huge increase in the number of 25(OH)D serum concentration tests performed within 6 years, from 4854 in 2006 to 20,089 in 2012. In 2006, family doctors and specialists gave disposal for 25(OH)D testing with similar frequency while in 2012 orders from family doctors prevailed (65.8% vs. 34.2%) [24].

In our study, vitamin D self-supplementation was declared more often by doctors ongoing non-surgical specialization than those in the surgical path (82.8% vs. 75.7%, respectively). This could be explained by the differences in adherence to medical guidelines between medical and surgical doctors [21,25]. Another reason could be the specifics of work performed by internists or GPs for whom prevention strategies establish an important part of practice. In the study by Fallon et al. [26], most of the surveyed practicing doctors and nurses from UK (78%) used vitamin D supplements to manage known deficiency, however some inconsistency in dosing was observed. 

The vitamin is known to play a role in modulating the immune system on a range of levels [27]. Several studies have examined the impact of vitamin D on the course of COVID-19 infection both as prophylaxis and treatment. According to some researchers, it could potentially mitigate the risk of infections [28,29]. Some authors advocate that vitamin D supplementation might potentially be useful in reducing the risk of contracting respiratory tract viruses, e.g., coronavirus [30,31,32]. On the other hand, more conservative researchers conclude that even if anti-viral prophylactic properties are not well-proven, supplementation with commercially available vitamin D preparations would do no harm considering the established health benefits with little potential for overdosing [33,34]. Interestingly, a recent study by D’Avolio et al. [35] showed significantly lower 25(OH)D levels in patients PCR-positive for SARS-CoV-2 compared with COVID-19-negative patients (11.1 ng/mL vs. 24.6 ng/mL). At the same time, Murai et al. discourage from administrating high doses of vitamin D in cases of moderate-to-severe COVID-19 infections as a treatment regime [36]. Amin and Drenos do not find vitamin D neither protective nor therapeutic in case of SARS-CoV-2 infections [37]. Although there are no solid arguments in favour of anti-viral therapies specifically based on vitamin D, the significance of maintaining 25(OH)D optimal serum concentration levels for overall health and immunological balance stands strong.

Since vitamin D deficiency may lead to severe changes in the body homeostasis, it should not be overseen during medical check-ups and must be acted upon in each case. This requires basic tools and knowledge which we attempted to assess based on physicians’ responses to our questionnaire. In the study, the score on vitamin D knowledge scale had a mean value of 6.8 and almost 19% of respondents obtained the middle value of 7. In a group of 158 primary health-care physicians from Riyadh (Saudi Arabia) [38], over half of respondents showed good knowledge on vitamin D and 55% had positive attitude about taking action concerning deficiency. When ways of managing vitamin D deficits were analyzed, older physicians as well as non-Saudis were found to provide nutritional advice [38]. A study based on a group of over 500 students from a university in Nanjing (China) [39] demonstrated that two-thirds of the participants knew well sources of vitamin D. In a study of medical students from Pakistan [40], most had great knowledge in terms of importance of vitamin D and 93% were aware of the consequences of its deficiency. In our group of Polish medical doctors, significantly higher scores in vitamin D knowledge were obtained by women and respondents with completed or ongoing non-surgical specialization. Similarly, respondents supplementing vitamin D had better knowledge on vitamin D than those who do not self-administer it. 

According to the results published by Costa-Fernandes et al., health care professionals in northwest London (UK) had overall good knowledge on vitamin D deficiency management [41]. The study further demonstrated that 75% of pediatricians and 65% of the general practitioners correctly identified the maintenance and treatment doses of vitamin D. On the contrary, almost 33% and 40% of prescribing doctors from Khartoum (Sudan) had poor general knowledge in terms of vitamin D deficiency treatment and poor nutrition knowledge score concerning vitamin D, respectively. In addition, the authors observed that about half of all doctors had poor attitude scores towards vitamin D, meaning little inclination towards maintaining optimal 25(OH)D levels in their patients [42]. Safdar et al. who examined 529 medical doctors and students in Jeddah (Saudi Arabia) observed that less than one-third of physicians and one-fifth of trainees knew the correct dose of vitamin D. The overall mean knowledge scores on vitamin D deficiency in this group was higher in postgraduates compared to students (20.0 ± 5.5 vs. 15.9 ± 5.5, respectively) [43]. This shows that proper counseling on vitamin D offered to health-care providers would prove beneficiary at most stages of medical education and training. 

This study has some limitations. First, it involved a relatively small, selected group of doctors attending obligatory courses at the School of Public Health, the Centre of Postgraduate Medical Education in Warsaw (Poland). However, the courses were obligatory for all doctors in specialization training in Poland and our voluntary study participants represented different regions and healthcare institutions from all over the country. Secondly, supplementing vitamin D was assessed based on self-declared responses, and we were not able to verify whether the described behaviors match the patients’ medical records. There is no national electronic medical records system in Poland to make this verification possible. Thirdly, our study group included doctors from different medical fields-categorized by the authors team as surgical and non-surgical but more studies are needed to assess the vitamin D supplementation and recommendation patterns within particular specialties. Another limitation is that the survey did not distinguish between different types of vitamin D supplements, i.e., D3-cholecalciferol and D2-ergocalciferol. However, since in Poland the vastly preferred and commercially available medical products contain vitamin D3, it can be safely assumed that the respondents described their experience with cholecalciferol.

## 5. Conclusions

Most medical doctors in Poland did not supplement vitamin D or did it occasionally, and one-quarter tested 25(OH)D serum concentration. Most respondents did not routinely recommend supplementing vitamin D to their patients. The level of knowledge about vitamin D among medical doctors in Poland is average, and is related to gender, type of specialization, own experience in vitamin D supplementation, and recommending supplementation to others. Our study revealed that the level of knowledge affects physicians’ behaviors towards vitamin D supplementation, and determines the patterns of recommendation to patients, relatives, friends and healthy adults.

The study indicates that medical doctors in Poland would benefit from additional training on vitamin D, in order to limit the ever increasing deficiency of this compound in the Polish population. Additional training should be addressed in particular to male doctors, pursuing surgical specialty, who do not supplement the vitamin D themselves. This would be of benefit to patients as well as their own health. 

## Figures and Tables

**Table 1:** Demographic characteristics of the study group.

Variable, Parameter	Category or Unit	Total (*N* = 701)
Gender, *n* (%)	Female	502 (71.61)
	Male	199 (28.29)
Age, min-max, M ± SD	Year	26–61, 32.1 ± 5.3
Age groups, *n* (%)	26–29	292 (41.65)
	30–39	337 (48.07)
	40+	72 (10.27)
Place of residence, *n* (%)	peripheral village	11 (1.57)
	village near agglomeration	26 (3.71)
	town up to 50,000 residents	63 (8.99)
	town 51,000–200,000 residents	88 (12.55)
	town 201,000–500,000 residents	104 (14.84)
	city over 500,000 residents	409 (58.35)

M—mean, SD—standard deviation. Results are presented as n—number of responses (%).

**Table 2:** Professional characteristics of the study group.

Variable, Parameter	Category or Unit	Total (*N* = 701)
Length of work as a medical doctor, min-max, M ± SD	Year	1–32, 5.7 ± 5.0
Job seniority groups, *n* (%)	1–3	321 (45.79)
	4–7	222 (31.67)
	8+	158 (22.54)
Main place of work, *n* (%)	clinical hospital	350 (49.93)
	public hospital	256 (36.52)
	non-public hospital	6 (0.86)
	public outpatient clinic	38 (5.42)
	non-public outpatient clinic	25 (3.57)
	private practice	12 (1.71)
	research institute	13 (1.85)
	Sanatorium	1 (0.14)
Specialization status, *n* (%)	Completed	61 (8.70)
	Ongoing	640 (91.30)
Type of specialization, *n* (%)	Surgical	202 (28.82)
	non-surgical	442 (63.05)
	both surgical and non-surgical	7 (1.00)
	no data	50 (7.13)
Ph.D., *n* (%)	Yes	50 (7.13)

M—mean, SD—standard deviation. Results are presented as n—number of responses (%).

**Table 3:** Health characteristics of the study group.

Variable, Parameter	Category or Unit	Total (*N* = 701)
BMI groups, *n* (%)	normal weight	555 (79.19)
	Overweight	118 (16.83)
	Obesity	23 (3.28)
	no data	5 (0.71)
How often do you use the advice of another doctor, *n* (%)	less often than once a year	344 (49.07)
	once a year	222 (31.67)
	once every 3 months	86 (12.27)
	more often than once every 3 months	49 (6.99)
Number of hospitalizations in the past 3 years, *n* (%)	0	519 (74.04)
	1	141 (20.11)
	2	34 (4.85)
	3	2 (0.29)
	4	5 (0.71)
Under medical treatment, *n* (%)	No	565 (80.60)
Myocardial infarction or ischemic stroke in the past, *n* (%)	Yes	1 (0.14)

Results are presented as n—number of responses (%).

**Table 4:** Attitudes towards vitamin D supplementation.

Variable, Parameter	Category or Unit	Total (*N* = 701)
Do you take vitamin D supplements, *n* (%)	no and never	132 (18.83)
	Yes	568 (81.17)
If you take vitamin D supplements, how often, *n* (%)	Occasionally	307 (43.79)
	regularly in autumn and winter	165 (23.54)
	regularly all-year-round	97 (13.84)
What dose of vitamin D supplements do you take, *n* (%)	800 units per day	8 (1.14)
	1000 units per day	112 (15.98)
	2000 units per day	347 (49.50)
	4000 units per day	73 (10.41)
	7000 units per week	6 (0.86)
	30,000 units per month	6 (0.86)
	other dose	8 (1.14)
	no data	141 (20.11)
Have you tested 25(OH)D serum concentration, *n* (%)	Yes	174 (24.82)
Results of 25(OH)D serum concentration testing, *n* (%) ^1^	Normal	66 (37.93)
	Low	108 (62.07)

^1^ estimated for respondents who tested vitamin D serum concentration (*N* = 174). Results are presented as n—number of responses (%).

**Table 5:** Practice in vitamin D supplementation.

Variable, Parameter	Category or Unit	Total (*N* = 701)
Do you recommend vitamin D supplements to relatives and friends, *n* (%)	Yes	577 (82.31)
Do you recommend vitamin D supplements to patients, *n* (%)	Yes	571 (81.46)
	No	124 (17.69)
	no data	6 (0.86)
If you recommend vitamin D supplements to patients, then in what situations, *n* (%)	only to patients with diagnosed deficiency	121 (17.26)
	only in autumn and winter	176 (25.11)
	to the majority of patients	274 (39.09)
Do you recommend vitamin D supplements to healthy adults, *n* (%)	Yes	592 (84.45)
	No	109 (15.55)
If you recommend vitamin D supplements to healthy adults, then in what dose, *n* (%)	1000 units per day	162 (23.11)
	2000 units per day	375 (53.50)
	4000 units per day	39 (5.56)
	other dose	16 (2.28)
Do you recommend vitamin D supplements to adults with diagnosed deficiency, *n* (%)	Yes	625 (89.16)
	No	29 (4.14)
	no data	47 (6.70)
If you recommend vitamin D supplements to adults with diagnosed deficiency, then in what dose, *n* (%)	1000 units per day	24 (3.42)
	2000 units per day	173 (24.68)
	4000 units per day	352 (50.21)
	7000 units per day	25 (3.57)
	30,000 units per week	20 (2.85)
	other dose	31 (4.42)

Results are presented as n—number of responses (%).

**Table 6:** Logistic regression analysis of not recommending vitamin D supplements to patients.

Covariate	Category or Unit	Univariate Model		Multivariate Model	
		OR	*p*	OR	*p*
Gender	male	1.611	0.022	1.030	0.923
	female	reference	-	reference	-
Age groups	<30	0.970	0.882	0.907	0.718
	30+	reference	-	reference	-
Type of specialization	surgical	1.956	0.001	2.214	0.004
	non-surgical	reference	-	reference	-
Do you take vitamin D supplements	no	2.774	<0.001	1.618	0.197
	yes	reference	-	reference	-
Do you recommend vitamin D supplements to relatives and friends	no	4.808	<0.001	2.137	0.033
	yes	reference	-	reference	-
Do you recommend vitamin D supplements to healthy adults	no	13.973	<0.001	4.664	<0.001
	yes	reference	-	reference	-
Do you recommend vitamin D supplements to adults with diagnosed deficiency	no	47.656	<0.001	12.255	<0.001
	yes	reference	-	reference	-

OR—odds ratio.

**Table 7:** Vitamin D knowledge in the study group.

Variable, Parameter	Category or Unit	✓ Correct Answer − Incorrect Answer	Total (*N* = 701)
Reasons for common, growing vitamin D deficiency are: (multiple answers)	Lifestyle, including spending time indoors	✓	681 (97.15)
	Reducing fats in diet	✓	203 (28.96)
	Common use of statins	✓	47 (6.70)
	Air pollution, including smog	✓	184 (26.25)
	Common use of sunscreen with UV filter	✓	235 (33.52)
	Obesity	✓	307 (43.79)
Groups which in particular require vitamin D supplementation are, apart from newborns and infants: (multiple answers)	Children and adolescents up to 18 years of age	✓	457 (65.19)
	Young adults	✓	186 (26.53)
	Pregnant and breastfeeding women	✓	583 (83.17)
	Overweight or obese persons	✓	385 (54.92)
	Persons older than 75 years	✓	556 (79.32)
Vitamin D poisoning is a very rare complication of supplementation or treatment with cholecalciferol pharmaceutical preparations and in fact it only affects people with genetically determined hypersensitivity to vitamin D (one answer)	yes	✓	426 (60.77)
	no	−	258 (36.80)
	I don’t know	−	17 (2.43)
Vitamin D poisoning is a common complication of treatment with active vitamin D metabolites, e.g., alfacalcidol (one answer)	yes	−	184 (26.25)
	no	✓	496 (70.76)
	don’t know	−	7 (1.00)

Results are presented as n—number of responses (%).

## Data Availability

The data presented in this study are available on request from Wojciech Stefan Zgliczyński. The data are not publicly available due to privacy restrictions.
